# Weight Loss Trajectories in Healthy Weight Coaching: Cohort Study

**DOI:** 10.2196/26374

**Published:** 2022-03-09

**Authors:** Sakris K E Kupila, Mikko S Venäläinen, Laura-Unnukka Suojanen, Milla Rosengård-Bärlund, Aila J Ahola, Laura L Elo, Kirsi H Pietiläinen

**Affiliations:** 1 Obesity Research Unit, Research Program for Clinical and Molecular Metabolism Faculty of Medicine University of Helsinki Helsinki Finland; 2 Turku Bioscience Centre University of Turku and Åbo Akademi University Turku Finland; 3 Abdominal Center, Obesity Center, Endocrinology University of Helsinki and Helsinki University Central Hospital Helsinki Finland; 4 Abdominal Center, Nephrology University of Helsinki and Helsinki University Central Hospital Helsinki Finland; 5 Folkhälsan Institute of Genetics Folkhälsan Research Center Helsinki Finland

**Keywords:** acceptance and commitment therapy, body weight, eHealth, obesity management, real-life intervention, web-based, mobile phone

## Abstract

**Background:**

As global obesity prevalence continues to increase, there is a need for accessible and affordable weight management interventions, such as web-based programs.

**Objective:**

This paper aims to assess the outcomes of healthy weight coaching (HWC), a web-based obesity management program integrated into standard Finnish clinical care.

**Methods:**

HWC is an ongoing, structured digital 12-month program based on acceptance and commitment therapy. It includes weekly training sessions focused on lifestyle, general health, and psychological factors. Participants received remote one-on-one support from a personal coach. In this real-life, single-arm, prospective cohort study, we examined the total weight loss, weight loss profiles, and variables associated with weight loss success and program retention in 1189 adults (963 women) with a BMI >25 kg/m² among participants of the program between October 2016 and March 2019. Absolute (kg) and relative (%) weight loss from the baseline were the primary outcomes. We also examined the weight loss profiles, clustered based on the dynamic time-warping distance, and the possible variables associated with greater weight loss success and program retention. We compared different groups using the Mann-Whitney test or Kruskal-Wallis test for continuous variables and the chi-squared test for categorical variables. We analyzed changes in medication using the McNemar test.

**Results:**

Among those having reached the 12-month time point (n=173), the mean weight loss was 4.6% (SE 0.5%), with 43% (n=75) achieving clinically relevant weight loss (≥5%). Baseline BMI ≥40 kg/m² was associated with a greater weight loss than a lower BMI (mean 6.6%, SE 0.9%, vs mean 3.2%, SE 0.6%; *P*=.02). In addition, more frequent weight reporting was associated with greater weight loss. No significant differences in weight loss were observed according to sex, age, baseline disease, or medication use. The total dropout rate was 29.1%. Dropouts were slightly younger than continuers (47.2, SE 0.6 years vs 49.2, SE 0.4 years; *P*=.01) and reported their weight less frequently (3.0, SE 0.1 entries per month vs 3.3, SE 0.1 entries per month; *P*<.001).

**Conclusions:**

A comprehensive web-based program such as HWC is a potential addition to the repertoire of obesity management in a clinical setting. Heavier patients lost more weight, but weight loss success was otherwise independent of baseline characteristics.

## Introduction

Global increases in the prevalence of obesity represent a pressing public health concern. Obesity is closely linked to various physical and mental health impairments, including type 2 diabetes, hypertension, nonalcoholic fatty liver disease, cancer, depression, and a decreased health-related quality of life, to name a few [1]. Among individuals with obesity, a moderate weight loss of 5% carries significant metabolic health benefits [2]. Furthermore, the etiology of obesity is multifactorial and consists of genetic, environmental, and behavioral factors. Behavioral factors, such as appetite control, eating habits, and physical activity, remain central targets in clinical practice and substantially impact treatment outcomes [3].

Treating obesity traditionally relies on lifestyle modifications during individual or group face-to-face sessions [4]. With ever-increasing rates of obesity and limited financial resources within health care systems, we must identify effective weight loss interventions that can be delivered to the wider public at a reasonable cost. Using information technology in obesity treatment may offer a cheaper alternative to relying on employed staff alone. Furthermore, while setting up an internet-based program may be expensive, upkeep can be cost-effective [5,6]. In addition, remote guidance may also reduce the number of necessary in-person consultations and minimize the travel time and costs associated with location-dependent treatment. Financial benefits and technology-based programs can be accessible regardless of the time of day, participants’ life situations, places of residence, and potential disabilities or oral communication difficulties [7].

Common successful components of web-based interventions include self-monitoring, professional feedback, goal-setting, social support, and a structured program [5,8-10]. Tailoring programs in this manner may enhance self-efficacy, evoke a feeling of being cared for, improve end results, increase engagement, and decrease attrition [11-14]. Furthermore, web-based programs may enhance the feeling of control over one’s own care and facilitate patient–expert and patient–patient interactions [11,15].

The effectiveness of web-based weight loss and weight management programs can diminish because of the poor use of the program and its elements. Reasons for this may include outdated website design, insufficient internet skills, limited internet connection, and patient unfamiliarity with web-based interventions in health care [15-17]. Moreover, users may perceive programs requiring self-monitoring to be burdensome [13]. Additional obstacles in technology-assisted programs may include problems with confidentiality and privacy, a decrease in face-to-face communication, medical errors caused by system malfunctions, technological errors, and data manipulation and misinterpretation, as well as legal, ethical, and administrative barriers [13,17]. Finally, high attrition rates and decreasing engagement over time represent common features of both eHealth innovations and weight loss interventions in general [18-20].

Although obesity treatment remains multidisciplinary, previously published studies typically focused on a limited number of factors important to successful weight loss. Most available studies on web-based programs offered no real-time human support to participants, and only a few studies have been conducted in real-life clinical settings in diverse patient groups. Moreover, previous studies have primarily examined small populations with short intervention and follow-up periods [21,22]. However, there are a few important exceptions. In a previous Danish study conducted in a real-life municipal setting, an eHealth intervention resulted in a significant weight reduction of 4.3% at the mean 7.3-month time point [23]. In patients who remained in the intervention for over 9 months, the mean weight loss was 6.3% [23]. Thus, although eHealth interventions are promising alternatives to conventional interventions, it remains important to assess their benefits in actual clinical care.

In this paper, we report our initial observations from a large cohort of patients taking part in a web-based platform, HealthyWeightHub, through which patients use an interactive program, Healthy Weight Coaching (HWC), for obesity management. The cohort consisted of men and women with a wide age and BMI range. Furthermore, the program relies on a broad spectrum of approaches (eg, diet, physical activity, sleep, health, psychological factors, and coping with stress) in the form of web-based training modules supported by a personal coach. In this real-life, single-arm, prospective cohort study, we assessed the outcomes of the HWC program. In doing so, we focused on the amount of weight loss, interindividual variability in the weight loss response, and possible variables associated with weight loss success and program retention.

## Methods

### Intervention

HWC is an ongoing 12-month interactive web-based intervention for weight management among patients identified with overweight or obesity. The program is available free of charge to all Finnish citizens as a part of Finnish public health care. In the program, we addressed a broad spectrum of health behaviors related to weight management, including diet, physical activity, sleep, psychological factors, coping with stress, and general health status. The program structure relied on weekly training sessions, and participants can freely choose from 200 available sessions to best meet their individual needs. Participants could submit daily—and were instructed to do so at least weekly—their weight, targets, feelings, diet, and physical activity logs to the program. In addition, each patient was assigned a personal coach (a nurse, nutritionist, physiotherapist, or psychologist) who offered remote one-on-one support and could individually tailor specific sessions to patients. One-on-one coaching was arranged biweekly during the first month, after which it was arranged monthly. Alongside coaching, participants were able to exchange messages with the coach whenever needed. Participants could also interact with each other using anonymous group chats. The group chats were arranged monthly. To ensure cybersecurity, the program required strong authentication.

The HWC program and its training sessions are based on the framework of acceptance and commitment therapy (ACT) and other theories of behavior change. ACT is a form of cognitive behavioral therapy that supports flexible decision-making in everyday life. Specifically, ACT increases mindfulness, self-regulation, and psychological flexibility [24-27]. In addition, the elements incorporated include self-monitoring, counselor feedback and communication, group support, a structured program, and individual tailoring, all of which appear to support successful weight loss [14,28,29]. The program has been previously described in detail [30].

### Participants

Patients entered the program based on a referral from a licensed physician in Finland. Most of the patients were from the Hospital District of Helsinki and Uusimaa. The general inclusion criteria in the referral process for the HWC were as follows: (1) age ≥18 years, (2) BMI ≥25 kg/m², (3) access to a computer or a smartphone with a stable internet connection, and (4) a willingness and motivation to participate in a web-based treatment program. Persons who were pregnant, lactating, or for other reasons required face-to-face treatment modalities were not accepted to the program, as their intervention would be conducted more safely in the clinics rather than via the internet.

### Ethical Considerations

Each participant provided written informed consent. The study was approved by the Coordinating Ethics Committee of the Helsinki University Hospital (reference number 327/13/03 /00/2015).

### Data Collection

The database for this study was created on March 12, 2019, and included data from all consenting participants since the initiation of the HWC in October 2016 [30]. To avoid confounding factors, we excluded participants who were diagnosed with type 1 diabetes; were following a very low-calorie diet; were taking weight loss medication; had undergone a gastric balloon procedure or bariatric surgery; and those who did not complete a 2-week trial period (n=264, 18.2%). All other patients who were initially referred to the HWC were included in the analyses.

We obtained data regarding age and sex from the Finnish national register. During the program, the participants completed several internet-based questionnaires, as previously described in detail [30]. To summarize, height and weight as well as baseline morbidities and medications were reported upon entry into the program. Daily reporting for weight was optional. However, at a minimum, weekly reports were encouraged as part of the training sessions. We used these weekly reports to interpolate the daily weight measurements. We calculated BMI as weight in kilograms divided by height in meters squared (kg/m²) and dichotomized it into groups with BMI <40 kg/m² (n=557) and BMI ≥40 kg/m² (n=631). This grouping at the morbid obesity cut-off point was used instead of conventional overweight (BMI 25-29.9 kg/m²) and obesity (BMI ≥30 kg/m²) groups, as only 25 individuals were identified in the overweight group. We also calculated the entry rate (entries per month) from the total number of weight entries divided by the time until the last weight entry. We considered participants who were inactive for >90 days as dropouts from the program.

### Statistical Analyses

As recruitment to the program remains continuous, the number of participants reaching each time point varied (Multimedia Appendix 1, Table S1). Unless otherwise stated, the figures and statistical analyses were based on the data from all participants. Data are presented as frequencies (%) for categorical variables and mean (SE) and median (IQR) for continuous variables. All continuous variables had skewed distributions (Shapiro-Wilk normality test). All statistical analyses were performed using the R statistical computing environment (version 3.4.1; R Foundation for Statistical Computing) [31]. Group comparisons were conducted using the Mann-Whitney test (2 groups) or Kruskal-Wallis test (>2 groups) for continuous variables and the chi-squared test for categorical variables. Changes in medication between baseline and 12 months were analyzed using the McNemar test. Weight loss patterns were clustered based on the dynamic time-warping distance and agglomerative hierarchical clustering using the R package *dtwclust* [32]*.* The R package *ggplot2* was used for the visualization of all results [33]. We set the level of significance at *P*<.05.

## Results

### Baseline Characteristics

Table 1 summarizes the baseline characteristics of the 1189 participants in this study. The mean age of patients was 48.6 (SE 0.3) years. BMI ranged from 26.3 to 78.7 kg/m^2^ (mean 40.6, SE 0.2 kg/m^2^). There were no statistically significant differences in age or BMI between men and women.

In total, 1044 (87.8%) participants reported comorbidities of some type. The mean number of comorbidities reported per person was 4.8 (SE 0.1; range 0-15). Hypertension was the most prevalent comorbidity (n=598, 57.2%), followed by dyslipidemia (n=368, 35.2%), allergies (n=368, 35.2%), sleep apnea (n=358, 34.2%), depression (n=343, 32.8%), and osteoarthritis or osteoarthrosis (n=318, 30.4%; Multimedia Appendix 1, Table S2). In addition, 18.5% (n=193) of the participants reported type 2 diabetes as a comorbidity.

Altogether, 1000 (84.1%) participants reported using any medication. The reported medications were grouped as shown in Multimedia Appendix 1, Table S3. The mean number of these medication groups per patient was 2.5 (SE 0.1, range 0-10). The most prevalent medication groups were cardiovascular drugs (n=513, 43.1%), followed by dietary supplements (n=434, 36.5%), metabolic and endocrine drugs (n=349, 29.4%), acute pain medication (n=348, 29.3%), and psychopharmacological drugs (n=284, 23.9%; Multimedia Appendix 1, Table S3). At 12 months (n=173), the mean number of medication groups remained the same (2.5, SE 0.03), whereas the use of respiratory and acute pain medication changed significantly (Multimedia Appendix 1, Table S4).

**Table 1:** Basic characteristics of the study population.

Characteristic	All (N=1189)			Women (n=963)			Men (n=226)	
	Value, mean (SE; range)	Value, median (IQR)	Value, mean (SE; range)		Value, median (IQR)	Value, mean (SE; range)		Value, median (IQR)
Age (years)	48.6 (0.3; 19.0-78.0)	50.0 (17.0)	48.4 (0.4; 19.0-78.0)		50.0 (17.0)	49.6 (0.8; 22.0-73.0)		52.0 (19.0)
Height (cm)	168.7 (0.2; 147.0-198.0)	168.0 (10.0)	166.0 (0.2; 147.0-184.0)		166.0 (8.0)	180.3 (0.5; 158.0-198.0)		180.0 (10.0)
Weight (kg)	115.8 (0.7; 60.2-284.0)	112.5 (30.2)	111.3 (0.7; 60.2-193.0)		108.0 (26.2)	135.2 (1.8; 83.0-284.0)		130.0 (32.2)
BMI (kg/m^2^)	40.6 (0.2; 26.3-78.7)	39.6 (8.3)	40.4 (0.2; 26.3-72.5)		39.4 (8.2)	41.5 (0.5; 27.2-78.7)		40.2 (8.6)

### Mean and Categorical Weight Loss

**Figure 1:**
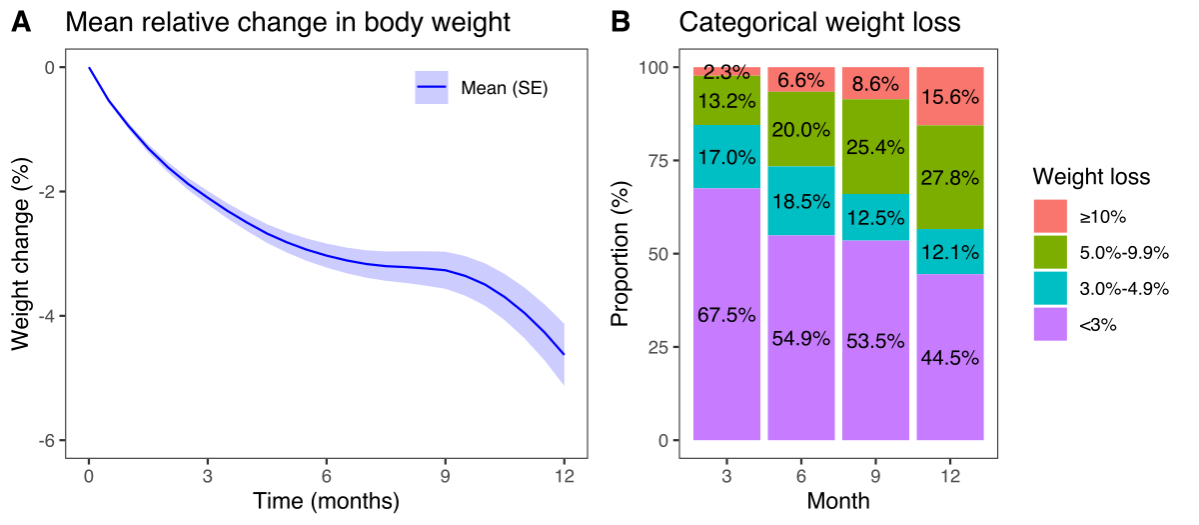
(A) Mean weight change (percentage from baseline) through the intervention; (B) distribution of weight loss categories at 3 (n=839), 6 (n=563), 9 (n=359), and 12 (n=173) months. The numbers indicate participants who have reached each time point.

The mean weight loss trajectory throughout the 12-month intervention period is shown in Figure 1A. At 12 months, the mean weight loss reached 4.6% (SE 0.5%) or 5.6 kg (SE 0.7 kg). The mean relative (%) and absolute (kg) weight losses at 3, 6, 9, and 12 months are shown in Multimedia Appendix 1, Table S5. Figure 1B shows the categorical weight loss at each time point. Among all participants who reached the 12-month time point by the data lock (n=173), 12.1% (n=21) lost 3% to 4.9%, 27.7% (n=48) lost 5% to 9.9%, and 15.6% (n=27) lost ≥10% of their baseline body weight. Altogether, 43.3% (n=75) of participants reaching the 12-month time point reported a clinically relevant weight loss of ≥5% of their initial weight.

At 12 months (n=173), the mean weight loss was similar in men (mean 5.4%, SE 1.4%) and women (mean 4.5%, SE 0.6%; Multimedia Appendix 1, Table S6). Participants aged <40 years achieved a weight loss (mean 4.3%, SE 1.2%) comparable with that of older individuals (mean 4.7%, SE 0.6%). Participants with a baseline BMI ≥40 kg/m² exhibited greater weight loss than those with a baseline BMI <40 kg/m² (mean 6.6%, SE 0.9% vs mean 3.2%, SE 0.5%; *P*=.02). Instead, patients with and without type 2 diabetes achieved similar weight loss rates (mean 5.3%, SE 1.2% vs mean 4.2%, SE 0.6%; *P*=.45, respectively). By the end of the intervention, patients who reported their body weight ≥4 times per month did not achieve statistically significantly greater weight loss (mean 5.9%, SE 1.1%) than those who reported a body weight <4 times per month (mean 4.2%, SE 0.6%; *P*=.09). However, at 3-, 6-, and 9-month time points, significantly greater weight loss was observed for patients who reported their body weights more frequently (*P*<.001).

### Interindividual Variation in Weight Loss Responses

**Figure 2:**
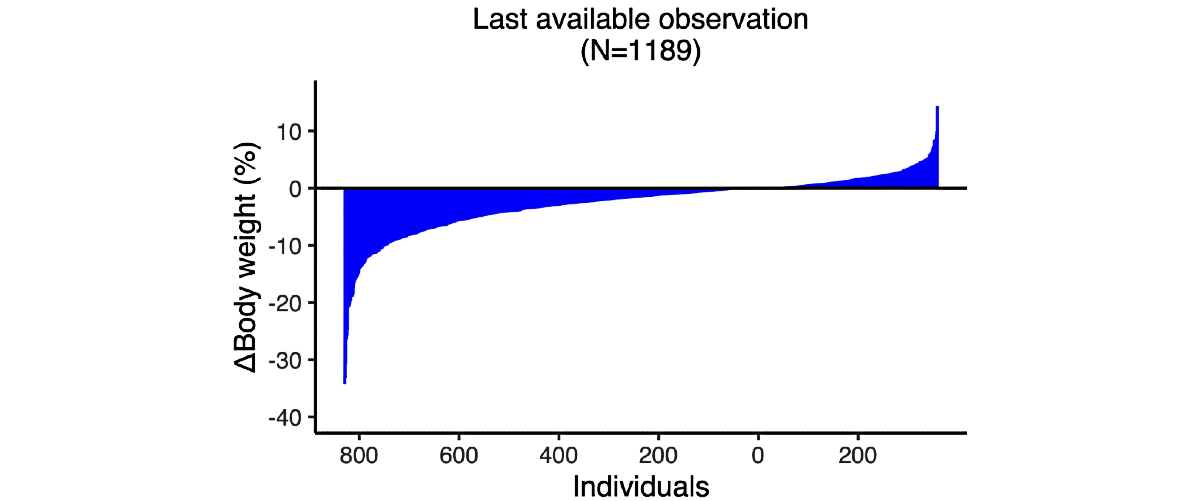
Individual weight change (percentage from baseline) at the last available time point.

In our attempt to delineate the interindividual variation in weight loss responses, we first plotted the last available observation from each participant, observing a wide range of weight changes, from −34.3% to +14.4% (Figure 2). Similarly, wide variations were observed at 3, 6, 9, and 12 months (Multimedia Appendix 1, Figure S1).

**Figure 3:**
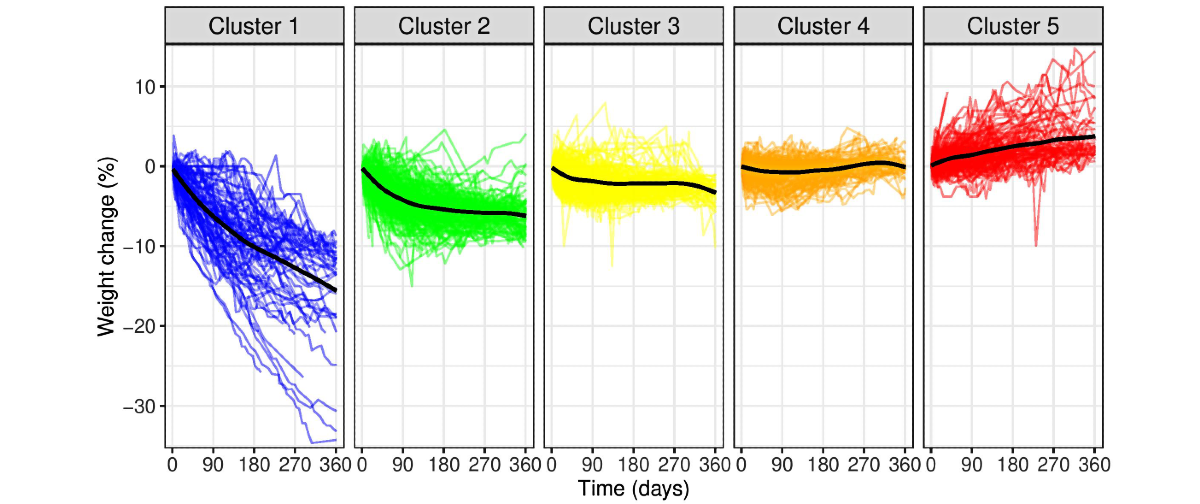
Clusters of study participants (N=1189) based on weight loss success trajectories. Individual weight loss patterns were clustered based on dynamic time-warping distance and agglomerative hierarchical clustering. Each colored line represents the weight change trajectory of a participant. The black line represents the average weight change in said cluster. Cluster 1, superresponders; cluster 2, responders; cluster 3, moderate responders; cluster 4, nonresponders; cluster 5, gainers.

We next analyzed the variation in weight changes using a dynamic time-warping algorithm and hierarchical clustering. In this process, we identified 5 clusters into which participants could be divided based on their weight loss success (Figure 3).

The mean weight changes in each of the 5 weight loss clusters from baseline to 3, 6, 9, and 12 months are shown in Multimedia Appendix 1, Table S7. Those in cluster 1 (superresponders; n=93, 8% of the study population) lost weight rapidly and consistently until the end of the 12-month period. In this group, participants lost an average of 15.7% (SE 1.3%) of their baseline body weight at 12 months.

In cluster 2 (responders; n=208, 17.5% of the study population), participants lost weight rapidly during the first 3 months, after which the weight loss rate slowed down. In this group, the participants had lost an average of 6.1% (SE 0.3%) of their baseline body weight at 12 months.

In cluster 3 (moderate responders; n=332, 27.9% of the study population), participants exhibited a small and steady mean 12-month weight loss of 3.4% (SE 0.3%) of their baseline body weight.

In cluster 4 (nonresponders; n=384, 32.3% of the study population), neither significantly lost nor gained weight. In this group, participants had lost on average 0.1% (SE 0.2%) of their baseline body weight at 12 months.

In cluster 5 (gainers; n=172, 14.5% of the study population), participants slowly gained weight rather than losing it. In this group, participants gained on average 3.5% (SE 0.6%) of their baseline body weight at 12 months.

Across all participants, 53.2% (n=633) fell into the category of responders (clusters 1-3), 32.3% (n=384) represented nonresponders (cluster 4), and 14.5% (n=172) represented gainers (cluster 5).

### Factors Explaining the Interindividual Variation in Weight Loss Response

To identify factors contributing to weight loss success, we analyzed whether the clusters differed according to age, sex, baseline BMI, concomitant diseases or medication use, or the frequency of weight entries. The clusters exhibited no statistically significant differences in age or sex (Multimedia Appendix 1, Table S8). Instead, a higher baseline BMI was associated with greater mean weight loss success (*P*<.001). Furthermore, greater mean weight loss was associated with more frequent weight entries (*P*=.004). In addition, the clusters differed only in relation to the number of medications (*P*=.048) but not in the medication groups and diseases (Multimedia Appendix 1, Table S9 and Figure S2).

### Attrition

Finally, we analyzed the attrition rate and possible associated factors. The total dropout rate at 12 months was 29.1% (n=346; Multimedia Appendix 1, Figure S3). In addition, given the continuous recruitment, 56.6% (n=689) of participants had not yet reached the 12-month time point at the time of the data lock.

We found no significant differences in the baseline BMI (mean 41.0 kg/m^2^, SE 0. 4 kg/m^2^ vs mean 40.4 kg/m^2^, SE 0.2 kg/m^2^; *P*=.12) or sex (272, 79% vs 691, 82% women; *P*=.21) between those who prematurely discontinued the program and those who remained continuously active (Multimedia Appendix 1, Table S10). Those discontinuing were younger (mean 47.2 years, SE 0.6 years vs 49.2 years, SE 0.4 years; *P*=.01) and made fewer monthly weight entries (mean 3.0, SE 0.1 entries/month vs mean 3.3, SE 0.1 entries per month; *P*<.001) compared with those who adhered. On the basis of their last reported body weight, most individuals who dropped out, clustered either as nonresponders or gainers (Multimedia Appendix 1, Figure S4).

## Discussion

### Principal Findings

The data collected in the novel web-based weight management program, HWC, offered a unique perspective of real-time obesity management in a large population of individuals with a wide age and BMI range. They also enabled us to identify subgroups of patients with different weight trajectories and trends in weight loss success. Furthermore, with a large number of included modules (including diet, physical activity, sleep, psychological factors, stress, and general health), the HWC program encompasses the multidisciplinary nature of clinical weight management with a long intervention time. Importantly, our patients, on average, presented with morbid obesity, with several comorbidities and the use of various medications. HWC is embedded into standard Finnish clinical care, and thus represents real-life clinical patient material. However, although the data collection was preplanned alongside the routine delivery of care, one needs to bear in mind that this real-life study has no control group.

We observed a mean weight loss of 4.6% (SE 0.5%) or 5.6 kg (SE 0.7 kg) at the 12-month time point. In a meta-analysis of 15 personalized eHealth studies, Lau et al [21] reported a 2.8 kg (range 2.0 kg-3.5 kg) greater mean weight loss in the eHealth intervention group than in the control group. Similarly, in a systematic review, a 2.4 kg higher weight loss was observed in traditional face-to-face treatments compared with no or minimal interventions [34]. In a previous Finnish randomized controlled trial, patients treated only via a web-based behavioral weight loss program lost on average 1.2% (range 0.3% to 2.2%) of their baseline body weight in 12 months, whereas the group treated via both the program and cognitive behavioral group therapy lost on average 3.5% (range 2.1% to 4.8%) of their baseline body weight [35]. In this real-life study, we did not have a control group, whereby we were unable to assess how a similar patient population to our study would have fared longitudinally without any treatment. On the basis of previous longitudinal studies, the BMI in Finnish cohorts tends to either increase [36] or remain stable [37] over time. Furthermore, in a Swedish Obese Subjects trial, where participants had a similar mean BMI (40.1 kg/m^2^) as in our study, there was no weight change in the control group with usual care at the 12-month time point [38]. Thus, it seems likely that the mean 4.6% (SE 0.5%) weight loss we observed was a result of the HWC treatment. However, based on a previous randomized controlled trial [35], it is also possible that combining web-based treatment with face-to-face behavioral therapy would have been more effective than web-based treatment alone.

We identified 5 clusters based on weight loss success, when examining weight change trajectories, allowing us to explore characteristics predicting weight loss outcomes. The clusters were comparable in terms of age and sex. However, a higher baseline BMI was associated with greater weight loss success. We found no statistically significant differences in the number of comorbidities or medication groups among clusters, suggesting that medication use and concomitant diseases did not define features of weight loss success. Of note, cluster 1, which had the best weight loss success, also had the largest variability in the weight loss results, which is most likely because this cluster size was the smallest population. Moreover, this cluster had the highest baseline BMI values. The largest of the formed clusters, cluster 4, comprised individuals with no change in their body weight. Indeed, it is very typical that not all patients undergoing weight loss interventions are successful in losing weight [23,39]. Identifying individuals and the reasons for not responding would be important to better support these individuals in future interventions. Overall, 53.2% (n=633) of the participants fell into clusters 1 to 3, and thus experienced some weight loss. Moreover, the observed mean weight loss was 4.6% (SE 0.5%), which is comparable with or somewhat higher than that previously reported [21,34,35].

No difference in weight loss was observed between individuals with and those without type 2 diabetes. Interestingly, in previous weight loss studies, patients with diabetes exhibited poorer success than those without diabetes [40,41]. One reason might be that diabetes as a comorbidity can render weight loss more difficult and harder to maintain [40,42]. However, diabetes and its related comorbidities may result in greater adherence and motivation for weight loss [43]. Furthermore, these novel interactive approaches may be more engaging and empowering than traditional methods because web-based programs may enhance a feeling of self-control and accountability regarding one’s care [15].

We found that a higher number of weight entries was associated with greater weight loss, in agreement with previous findings [10,44,45]. We also found that those who dropped out made fewer weight entries to the program than did those who adhered. However, whether the number of entries attributes to success itself remains unclear. Success may make the program more rewarding, thus prompting a patient to log weights more frequently and engage more with the program. As for attrition, it is probable that individuals who fail to lose weight or experience any other benefits more readily drop out from the programs. In addition, discontinuing an intervention may also occur because the participants already met their personal goals before the targeted end date [17,46-48]. Understanding the nature of this relationship between success and the weight entry rate requires further investigation.

In the HWC program, we observed an attrition rate of 29.1% (n=346). Patients who dropped out were younger and more often belonged to the group that did not lose weight in the program. Retention and weight loss may be bidirectionally related to each other: patients retained in the program have more support for weight loss attempts, and better weight loss motivates them to remain active in the program. Overall, attrition may bias the outcome and should be considered when interpreting the results of the study. High attrition rates and decreasing engagement over time represent common features of both eHealth innovations and clinical face-to-face weight loss interventions [18-20]. This may be especially true in long-term interventions, such as this program. Similar to our study, the 2 eHealth platforms for weight management reported a 54% attrition rate in a clinical setting [35,46]. This is comparable with a traditional face-to-face clinic-based intervention, which also reported that 54% of patients discontinued [19]. Previous meta-analyses on eHealth-based weight loss interventions primarily focused on randomized controlled trials and determined average attrition rates close to 20% [6,10]. However, these rates might misrepresent attrition in real-life programs given inherent differences in, for example, participant demographics (ie, real clinical patients vs volunteers) and program design (ie, additional assessment sessions, participant retention strategies, and greater accountability).

This study had several strengths and limitations. An important strength of this study lay in its reliance on a large sample of individuals with a broad age and BMI distribution. With a large number of reported comorbidities and medications, our data provided a realistic view of treating patients with obesity-related complications. Although using data from a real-life clinical setting offers a tangible and practical view of the web-based management of obesity, the program was not randomized and was thus lacking a control group. Subsequently, the study has low internal validity, and we are unable to determine the efficacy of the program compared with no treatment, face-to-face treatment, or any other comparison. We were also not able to study those patients who needed treatment for obesity but were not willing to participate in a web-based program and received a referral to HWC in the first place. In addition, as body weight was self-reported, the possibility of misreporting will need to be considered when interpreting the results. It should also be noted that the program comprises several components. On the basis of the current analyses, it is not possible to distinguish between the different treatment effects, dose responses, or mechanisms of change in these components. The current report describes the overall outcomes of this program. Finally, as is the case with most previous studies, our participants consisted predominantly of women and adults of middle age. Therefore, our conclusions are not fully generalizable to younger adults and men.

Future research should focus on younger adults and men, in particular, and attempt to examine the effects and appeal of web-based programs. In addition, further investigation of the factors associated with successful weight loss and reasons for attrition would prove beneficial. For example, future studies should attempt to determine when attrition occurs because of reaching weight loss targets early instead of genuine nonuse. This would advance the development of targeted web-based programs tailored to specific patient subgroups.

### Conclusions

In conclusion, HWC is a web-based method for the management of obesity in real-life clinical settings. During the 12-month treatment, the average weight loss was 4.6% (SE 0.5%) but varied widely. Specifically, baseline medication use, baseline health status, age, or sex was not significantly associated with weight loss in the HWC program. Further research is needed to understand the determinants of weight loss success.

## Appendix

Multimedia Appendix 1 Supplementary tables and figures.

## Abbreviations

ACT: acceptance and commitment therapy
HWC: Healthy Weight Coaching
