# Supplementary material for: Weight Loss Trajectories in Healthy Weight Coaching: Cohort Study
Source: JMIR Form Res. 2022 Mar 9;6(3):e26374. doi: 10.2196/26374 (PMC8943569; doi:10.2196/26374)
Supplement: Multimedia Appendix 1 [file formative_v6i3e26374_app1.pdf]

Weight Loss Trajectories in Healthy Weight Coaching: Cohort Study

Sakris Kupila, BM[1]*; Mikko S Venäläinen, PhD[2]*; Laura U Suojanen, MSc[3]; Milla Rosengård-Bärlund, MD, PhD[3]; Aila J Ahola, PhD[1,3,4,5]; Laura L Elo, PhD[2]; Kirsi H Pietiläinen, MD, PhD[1,3]

[1]Obesity Research Unit, Research Program for Clinical and Molecular Metabolism, Faculty of Medicine, University of Helsinki, Finland

[2]Turku Bioscience Centre, University of Turku and Åbo Akademi University, Finland

[3]Abdominal Center, Obesity Center, Endocrinology, University of Helsinki and Helsinki University Central Hospital, Helsinki, Finland

[4]Department of Nephrology, University of Helsinki and Helsinki University Hospital, Helsinki, Finland

[5]Folkhälsan Institute of Genetics, Folkhälsan Research Center, Helsinki, Finland

* These authors contributed equally to this work

Corresponding author: Kirsi Pietiläinen, Obesity Research Unit, Biomedicum 1, Haartmaninkatu 8, P.O. Box 63, FI-00014 University of Helsinki, Finland. Telephone +358-505992295. E-mail: kirsi.pietilainen@helsinki.fi

Table S1. Distribution of the 1189 study participants across different time points in the 12-month program at the time of data analysis

Month	N
0	102
1	125
2	123
3	86
4	100
5	91
6	67
7	57
8	79
9	55
10	45
11	86
12	173
**Total**	**1189**

Table S2. Prevalence of diseases at baseline

Disease	N	Proportion of the study population (%)
no disease	145	12.2
hypertension	598	57.2
allergies	368	35.2
dyslipidaemia	368	35.2
sleep apnoea	358	34.2
depression	343	32.8
osteoarthritis or osteoarthrosis	318	30.4
headache	265	25.3
asthma	258	24.7
prediabetes	253	24.2
anxiety	204	19.5
type 2 diabetes	193	18.5
hypothyreosis	189	18.1
fatty liver	155	14.8
gallstones	136	13.0
heart arrhythmia	125	12.0
chronic back pain	116	11.1
surgery within the past year	97	9.3
other mental illness	85	8.1
binge eating disorder	75	7.2
diaphragmatic hernia	66	6.3
anaemia	64	6.1
gastroesophageal reflux disease	60	5.7
cancer	51	4.9
gout	47	4.5
other lung disease	25	2.4
other vascular disease	24	2.3
alcoholism	22	2.1
rheumatoid arthritis	21	2.0
inflammatory bowel disease	19	1.8
deep vein thrombosis	18	1.7
hyperthyreosis	17	1.6
coronary artery disease	17	1.6
peripheral artery disease	16	1.5
chronic obstructive pulmonary disease	14	1.3
ischemic stroke	11	1.1
myocardial infarction	11	1.1
valvular heart disease	9	0.9
cerebrovascular disease	8	0.8
anorexia	7	0.7
haemorrhagic stroke	2	0.2
memory disorder	1	0.1

Table S3. Prevalence of medication types at baseline

Medication type	N	Proportion of the study population (%)
no medication	189	15.9
cardiovascular	513	43.1
dietary supplements	434	36.5
metabolic and endocrine	349	29.4
acute pain	348	29.3
psychopharmacological	284	23.9
allergy	203	17.1
respiratory	190	16.0
gastrointestinal	171	14.4
sex hormones	123	10.3
sleep	91	7.7
neurological	88	7.4
other	75	6.3
corticosteroids and immunomodulators	54	4.5
chronic pain	32	2.7
urological	10	0.8
antimicrobial	1	0.1

Table S4. Prevalence of medication types at 12 months

Medication type		0 months	12 months		
		N	Yes (%)	No (%)	*P* value
cardiovascular	Yes	44	79.5	20.5	0.146
	No	52	5.8	94.2	
dietary supplements	Yes	46	58.7	41.3	0.736
	No	50	32.0	68.0	
metabolic and endocrine	Yes	30	66.7	33.3	0.454
	No	66	9.1	90.9	
acute pain	Yes	9	77.8	22.2	0.002
	No	87	17.2	82.8	
psychopharmacological	Yes	14	85.7	14.3	0.109
	No	82	9.8	90.2	
allergy	Yes	20	70.0	30.0	1.000
	No	76	9.2	90.8	
respiratory	Yes	17	52.9	47.1	0.039
	No	79	1.3	97.8	
gastrointestinal	Yes	16	62.5	37.5	1.000
	No	80	7.5	92.5	
sex hormones	Yes	10	20.0	80.0	0.503
	No	86	14.0	86.0	
sleep	Yes	12	33.3	66.7	0.791
	No	84	7.1	92.9	
neurological	Yes	4	75.0	25.0	1.000
	No	92	1.1	98.9	
corticosteroids and immunomodulators	Yes	3	0	100	0.727
	No	93	5.4	94.6	
chronic pain	Yes	3	0	100	NA
	No	93	0	100	
urological	Yes	1	0	100	1.000
	No	95	2.1	97.9	
antimicrobial	Yes	0	0	0	NA
	No	96	0	100	
other	Yes	6	16.7	83.3	0.143
	No	90	13.3	86.7	

The column “0 months” shows how many of the participants reaching 12 months reported using each medication type at baseline (yes = reports using said medication type). The column “12 months” shows the percentage of those participants using the same medication type at 12 months. McNemar test.

Table S5. Mean relative (%, standard error (SE), interquartile range (IQR)) and absolute (kg) weight changes at 3, 6, 9, and 12 months

		Weight change (%)		Weight change (kg)	
**Month**	**N**	**Mean (SE)**	**Median (IQR)**	**Mean (SE)**	**Median (IQR)**
3	839	-2.1 (0.1)	-1.6 (3.7)	-2.5 (0.1)	-1.8 (4.2)
6	563	-3.1 (0.2)	-2.4 (5.5)	-3.7 (0.3)	-2.9 (6.0)
9	359	-3.2 (0.3)	-2.6 (6.9)	-3.8 (0.4)	-2.9 (7.1)
12	173	-4.6 (0.5)	-4.0 (7.8)	-5.6 (0.7)	-4.1 (7.9)

Table S6. Mean 12-month weight loss (% from baseline) by sex, age, type 2 diabetes status, baseline body mass index and weight entry rate

			Relative weight change at 3 months (%)				
**Variable**		**N**	**Mean**	**SE**	**Median**	**IQR**	***P* value**
Sex	Women	704	-2.1	0.1	-1.7	3.6	0.633
	Men	135	-2.1	0.3	-1.4	4.4	
Age (years)	<40	195	-1.8	0.2	-1.4	3.3	0.135
	≥40	644	-2.1	0.1	-1.7	3.7	
Type 2 diabetes	No	645	-2.1	0.1	-1.7	3.7	0.763
	Yes	139	-2.0	0.2	-1.7	3.6	
Body mass index (kg/m^2)	<40	453	-1.8	0.1	-1.5	3.4	0.019
	≥40	386	-2.4	0.2	-1.8	3.7	
Weight entry rate	<4	653	-1.8	0.1	-1.4	3.3	<0.001
(entries/month)	≥4	186	-3.0	0.3	-2.6	4.0	
			**Relative weight change at 6 months (%)**				
**Variable**		**N**	**Mean**	**SE**	**Median**	**IQR**	***P* value**
Sex	Women	480	-3.0	0.2	-2.4	5.4	0.340
	Men	83	-3.7	0.6	-2.9	5.7	
Age (years)	<40	123	-2.4	0.4	-1.8	4.6	0.082
	≥40	440	-3.3	0.2	-2.7	5.7	
Type 2 diabetes	No	437	-3.0	0.2	-2.4	5.2	0.731
	Yes	89	-2.8	0.5	-2.2	4.9	
Body mass index (kg/m^2)	<40	301	-2.5	0.2	-2.4	5.5	0.055
	≥40	262	-3.7	0.3	-2.5	5.3	
Weight entry rate	<4	441	-2.5	0.2	-2.2	5.1	<0.001
(entries/month)	≥4	122	-5.1	0.5	-4.2	6.2	
			**Relative weight change at 9 months (%)**				
**Variable**		**N**	**Mean**	**SE**	**Median**	**IQR**	***P* value**
Sex	Women	307	-3.1	0.3	-2.6	6.7	0.460
	Men	52	-4.2	0.8	-2.7	7.1	
Age (years)	<40	69	-1.8	0.6	-1.2	7.0	0.028
	≥40	290	-3.6	0.3	-2.9	6.5	
Type 2 diabetes	No	280	-3.0	0.3	-2.5	6.5	0.475
	Yes	60	-3.9	0.7	-2.9	7.6	
Body mass index (kg/m^2)	<40	204	-2.5	0.3	-2.3	7.1	0.121
	≥40	155	-4.2	0.5	-2.8	6.2	
Weight entry rate	<4	280	-2.8	0.3	-2.1	6.4	<0.001
(entries/month)	≥4	79	-4.9	0.6	-4.5	6.8	
			**Relative weight change at 12 months (%)**				
**Variable**		**N**	**Mean**	**SE**	**Median**	**IQR**	***P* value**
Sex	Women	144	-4.5	0.6	-4.1	7.5	0.721
	Men	29	-5.4	1.4	-3.6	8.9	
Age (years)	<40	26	-4.3	1.2	-4.4	7.7	0.956
	≥40	147	-4.7	0.6	-4.0	7.7	
Type 2 diabetes	No	140	-4.2	0.6	-3.6	7.5	0.446
	Yes	26	-5.3	1.2	-4.5	9.0	
Body mass index (kg/m^2)	<40	101	-3.2	0.5	-2.8	7.7	0.016
	≥40	72	-6.6	0.9	-5.2	7.6	
Weight entry rate	<4	135	-4.2	0.6	-3.1	7.4	0.091
(entries/month)	≥4	38	-5.9	1.1	-4.9	6.1	

Table S7. Mean weight changes (% from baseline) in the weight loss clusters

	3 Months			6 Months			9 Months			12 Months		
**Cluster (baseline N)**	**N**	**Mean (SE)**	**Median (IQR)**	**N**	**Mean (SE)**	**Median (IQR)**	**N**	**Mean (SE)**	**Median (IQR)**	**N**	**Mean (SE)**	**Median (IQR)**
1 (93)	91	–6.3 (0.4)	-6.1 (5.2)	79	–10.3 (0.6)	-9.8 (7.0)	49	–12.7 (0.9)	-10.7 (7.3)	28	–15.7 (1.3)	-13.1 (7.2)
2 (208)	188	–4.2 (0.2)	-4.0 (2.8)	148	–5.4 (0.2)	-5.4 (3.0)	99	–5.9 (0.2)	-5.8 (1.9)	55	–6.1 (0.3)	-6.6 (2.7)
3 (332)	242	–1.9 (0.1)	-1.9 (1.8)	136	–2.2 (0.1)	-2.3 (1.7)	80	–2.2 (0.2)	-2.3 (1.5)	32	–3.4 (0.3)	-3.1 (1.9)
4 (384)	173	–0.8 (0.1)	-0.4 (1.3)	107	–0.4 (0.1)	-0.2 (1.8)	67	0.2 (0.2)	0.0 (1.6)	33	–0.1 (0.2)	0.0 (0.6)
5 (172)	145	1.4 (0.1)	1.4 (2.2)	93	2.5 (0.2)	1.9 (2.4)	64	3.2 (0.3)	2.1 (3.1)	25	3.5 (0.6)	2.2 (1.5)

SE, standard error; IQR, interquartile range. Individual weight loss patterns were clustered based on dynamic time-warping distance and agglomerative hierarchical clustering. We identified five clusters based on weight loss success. Cluster 1, super-responders; cluster 2, responders; cluster 3, moderate responders; cluster 4, non-responders; cluster 5, gainers.

Table S8. Age, sex, body mass index, and weight entry rate in the weight loss clusters

	Age (years)		Sex		BMI (kg/m^2)		Weight entries per month	
**Cluster (N)**	**Mean (SE)**	**Median (IQR)**	**Women (%)**	**Men (%)**	**Mean (SE)**	**Median (IQR)**	**Mean (SE)**	**Median (IQR)**
1 (93)	50.5 (1.2)	52.0 (16.0)	79.6	20.4	43.2 (0.9)	42.3 (10.4)	3.5 (0.2)	3.3 (2.2)
2 (208)	49.5 (0.8)	51.0 (16.3)	85.1	14.9	40.5 (0.5)	39.5 (8.7)	3.3 (0.1)	3.1 (1.9)
3 (332)	48.4 (0.6)	50.0 (18.0)	82.5	17.5	40.7 (0.4)	39.7 (8.3)	3.2 (0.1)	2.8 (2.3)
4 (384)	48.0 (0.6)	49.0 (18.0)	79.2	20.8	40.8 (0.4)	39.5 (7.8)	3.2 (0.1)	2.8 (2.2)
5 (172)	48.2 (0.9)	50.0 (16.0)	77.9	22.1	38.6 (0.5)	37.9 (7.4)	2.9 (0.2)	2.6 (1.8)
P	0.302		0.308		<0.001		0.004	

BMI, body mass index; SE, standard error; IQR, interquartile range. Kruskal-Wallis test for continuous variables, Chi-squared test for categorical variables.

Table S9. Number of diseases and medications in the weight loss clusters

	Cluster 1 (N=93)		Cluster 2 (N=208)		Cluster 3 (N=332)		Cluster 4 (N=384)		Cluster 5 (N=172)		
**Characteristic**	**Mean (SE)**	**Median (IQR)**	**Mean (SE)**	**Median (IQR)**	**Mean (SE)**	**Median (IQR)**	**Mean (SE)**	**Median (IQR)**	**Mean (SE)**	**Median (IQR)**	***P***
Number of diseases	4.9 (0.3)	4.0 (3.0)	4.7 (0.2)	4.0 (4.8)	4.8 (0.2)	4.0 (3.0)	4.9 (0.1)	5.0 (3.0)	4.8 (0.2)	5.0 (3.0)	0.796
Number of disease groups	4.0 (0.2)	4.0 (2.0)	3.9 (0.1)	4.0 (3.0)	3.8 (0.1)	4.0 (3.0)	3.8 (0.1)	4.0 (2.0)	3.7 (0.2)	3.0 (3.0)	0.561
Number of medications	3.4 (0.3)	3.0 (4.0)	3.4 (0.2)	3.0 (3.0)	3.3 (0.1)	3.0 (4.0)	3.0 (0.1)	3.0 (3.0)	3.5 (0.2)	3.0 (3.0)	0.048
Number of medication groups	2.5 (0.2)	3.0 (3.0)	2.6 (0.1)	2.0 (3.0)	2.5 (0.1)	2.0 (2.0)	2.3 (0.1)	2.0 (3.0)	2.7 (0.1)	2.5 (2.0)	0.100

SE, standard error; IQR, interquartile range. Kruskal-Wallis test.

Table S10. Comparison of those continuing the study to those dropping out based on baseline body mass index, age, monthly weight entry rate, and sex

**Characteristic**	**Continuers (N=843)**		**Dropouts (N=346)**		
	**Mean (SE)**	**Median (IQR)**	**Mean (SE)**	**Median (IQR)**	***P* value**
Body mass index (kg/m^2)	40.4 (0.2)	39.4 (8.1)	41.0 (0.4)	40.1 (8.6)	0.120
Age (years)	49.2 (0.4)	51.0 (17.0)	47.2 (0.6)	49.0 (18.0)	0.011
Monthly weight entry rate	3.3 (0.1)	3.0 (2.2)	3.0 (0.1)	2.5 (2.2)	<0.001
	**N**	**%**	**N**	**%**	
Women	691	82.0	272	78.6	0.208
Men	152	18.0	74	21.4	

SE, standard error; IQR, interquartile range. Mann-Whitney U-test for continuous variables, Chi-squared test for categorical variables.

Figure S1. Individual weight changes (% from baseline) at the 3, 6, 9, and 12-month time points

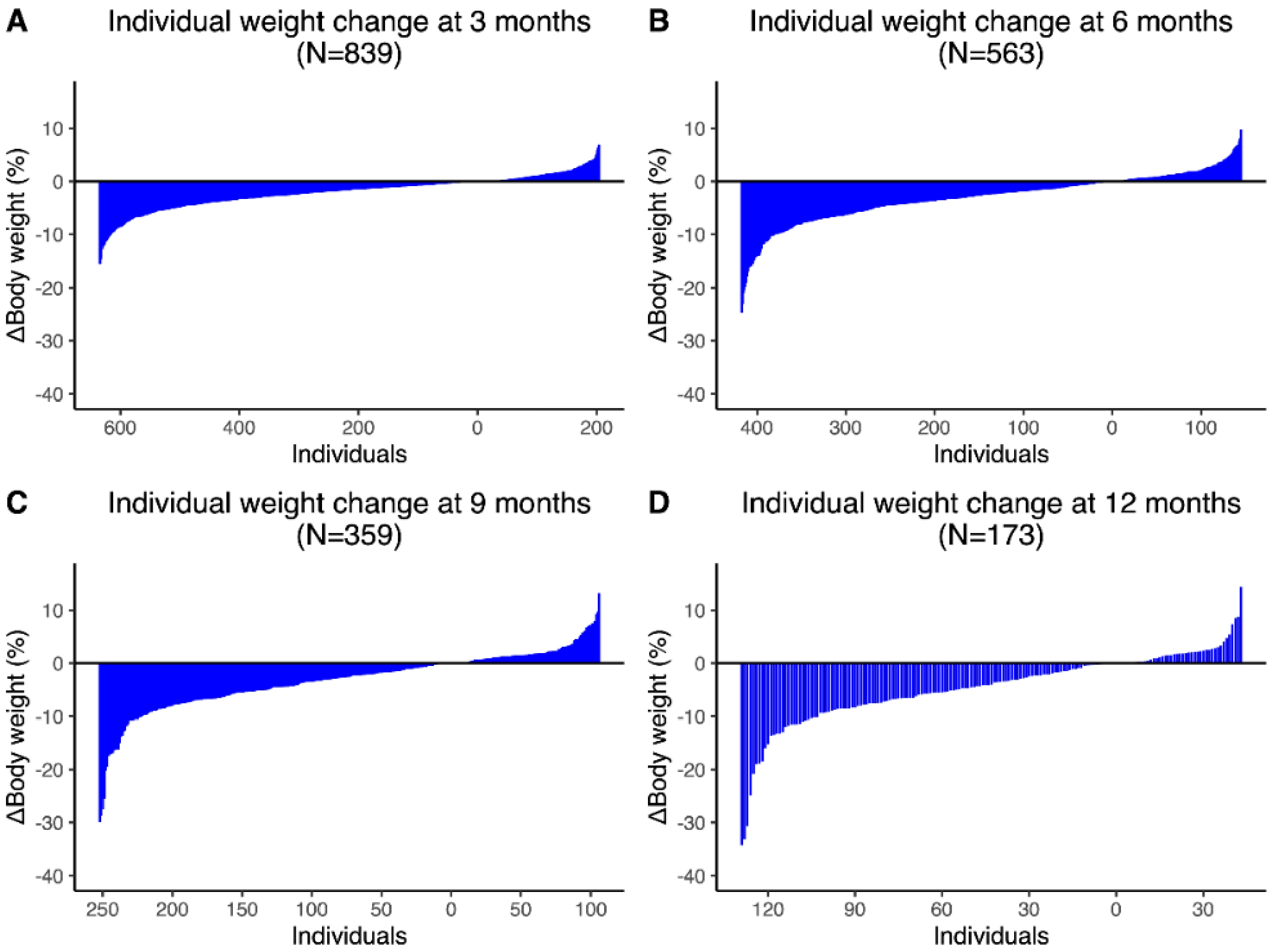

Figure S2. A) Disease groups among the weight loss clusters. B) Medication groups among the weight loss clusters.

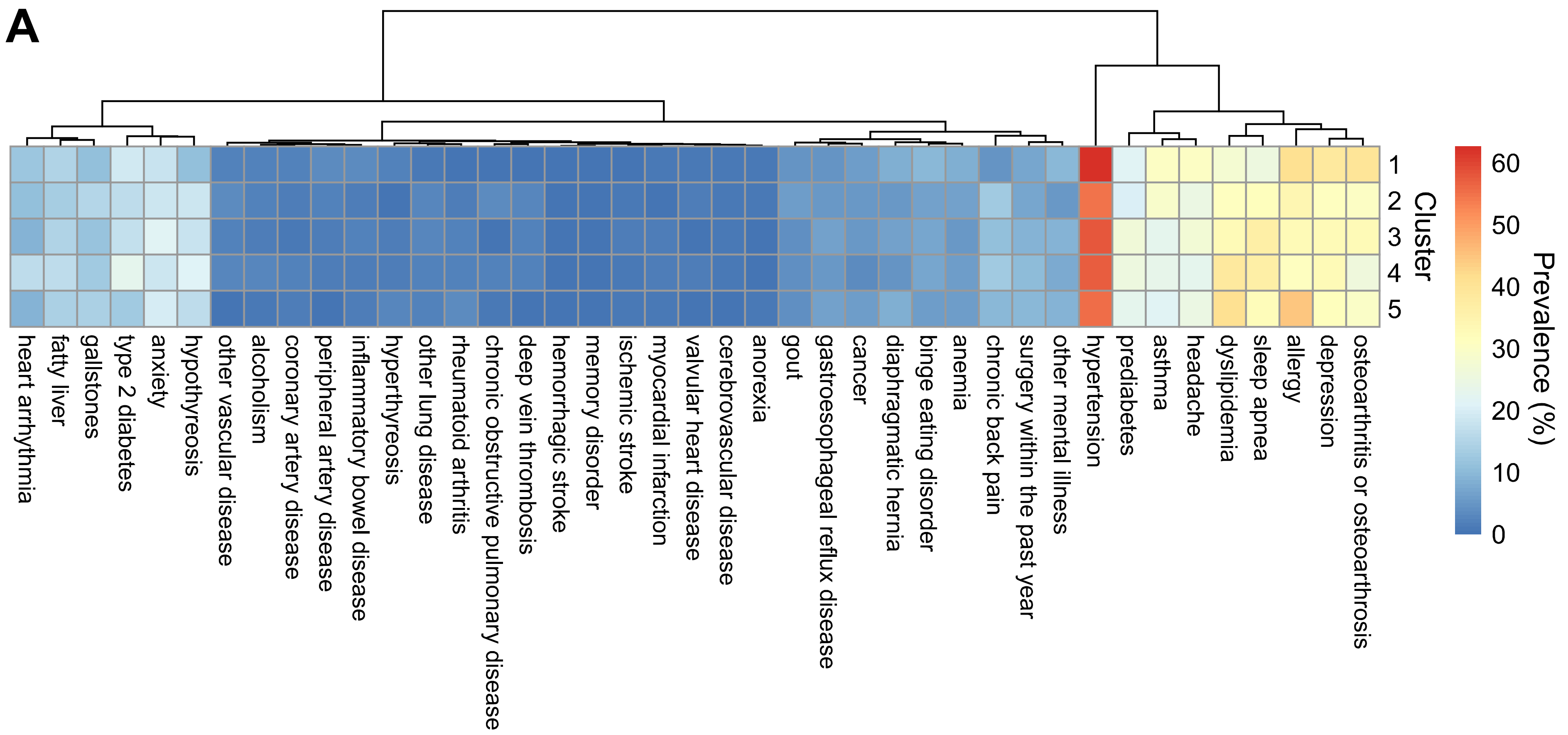

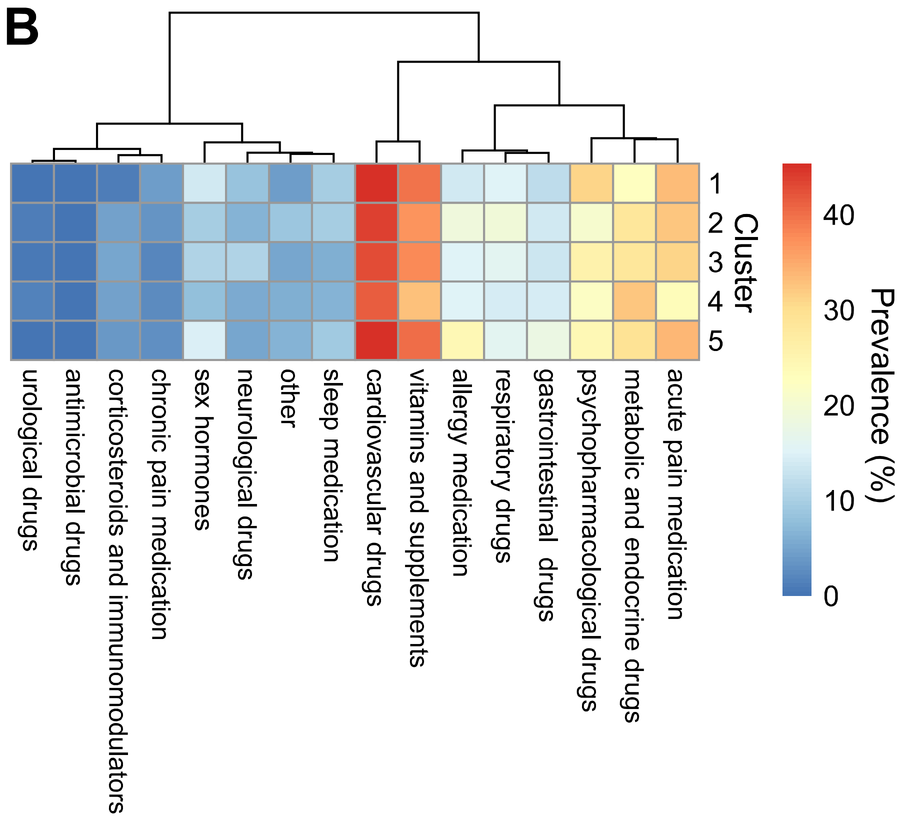

Individual weight loss patterns were clustered based on dynamic time-warping distance and agglomerative hierarchical clustering. We identified five clusters based on weight loss success. Cluster 1, super-responders; cluster 2, responders; cluster 3, moderate responders; cluster 4, non-responders; cluster 5, gainers.

Figure S3. Flow of patients in the 12-month program; patients still active, those having dropped out, and those having completed the program.

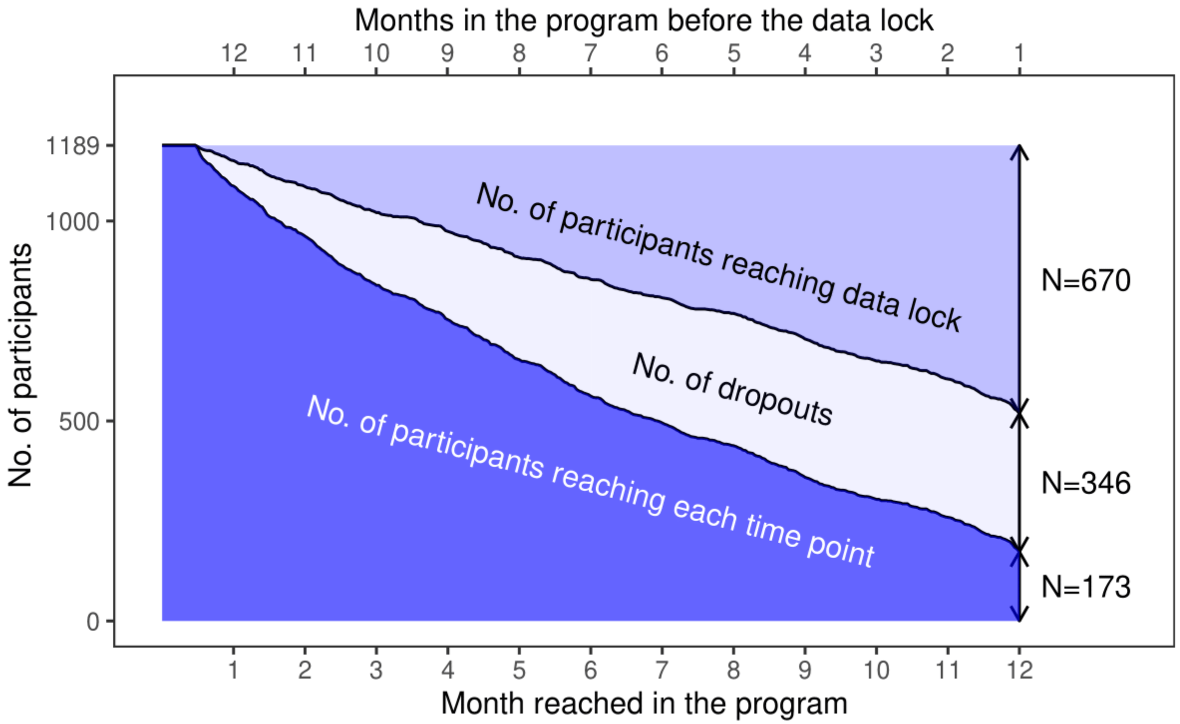

Figure S4. Proportion of dropouts in different weight loss success clusters.

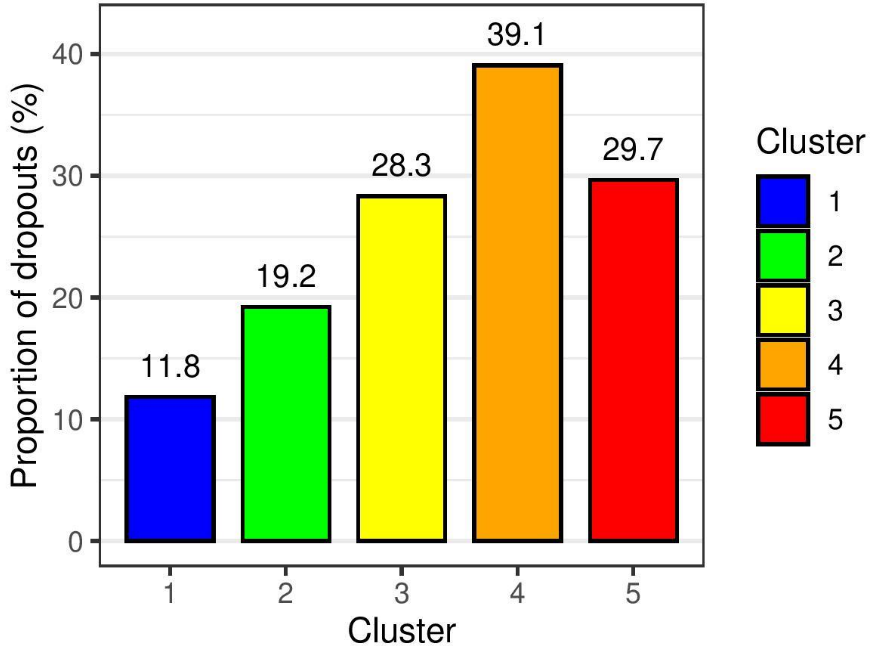

Individual weight loss patterns were clustered based on dynamic time-warping distance and agglomerative hierarchical clustering. We identified five clusters based on weight loss success: cluster 1, superresponders; cluster 2, responders; cluster 3, moderate responders; cluster 4, nonresponders; cluster 5, gainers. Participants inactive for >90 days were considered as dropouts from the program.
